# Hyper-converged space-air-ground integrated low-altitude aerial vehicular networks: Architecture, challenges, and technologies

**DOI:** 10.1016/j.fmre.2025.12.034

**Published:** 2026-02-26

**Authors:** Haibo Zhou, Jianzhe Xue, Yi Yuan, Zeyu Sun, Yunting Xu, Jiacheng Chen, Xuemin Shen

**Affiliations:** a School of Electronic Science and Engineering, Nanjing University, 210023 Nanjing, Jiangsu, China; b School of Computer Science and Engineering, Nanyang Technological University, 639798, Singapore; c Department of Strategic and Advanced Interdisciplinary Research, Peng Cheng Laboratory, 518000 Shenzhen, Guangdong, China; d Department of Electrical and Computer Engineering, University of Waterloo, N2L3G1 Waterloo, Ontario, Canada

**Keywords:** Low-altitude network, Space-air-ground, DOICT, 6G, Ubiquitous connectivity

## Abstract

In this paper, we introduce the space-air-ground integrated low-altitude aerial vehicular networks (SAG-LAAVN) that deliver ubiquitous intelligent connectivity for three-dimensional intelligent transportation systems (ITS). Built upon an innovative hyper-converged architecture that unifies Data, Operation, Information, and Communication Technologies (DOICT), SAG-LAAVN aims to address the stringent connectivity requirements imposed by the rapid evolution of low-altitude aerial vehicles, offering seamless coverage, enhanced resilience, and improved quality of experience across diverse scenarios. Three key scientific challenges in the SAG-LAAVN are discussed. Besides, we present three potential technologies: intelligent networking approach, multi-dimensional resource management, and virtual-physical dynamic collaboration. Implemented via the fully decoupled radio access network (FD-RAN), a case study is presented to demonstrate that SAG-LAAVN can provide ubiquitous, high-quality services that meet the evolving needs of the low-altitude economy.

## Graphical abstract

**Figure:**
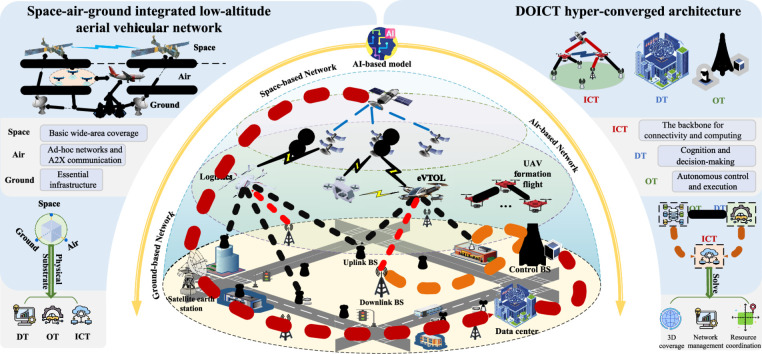

## 1 Introduction

Rapid advances in transportation and communication technologies facilitate the development of the low-altitude economy and urban air mobility (UAM), expanding traditional ground transportation into a three-dimensional form [1]. Driven by the need to reduce traffic congestion and improve transportation efficiency in complex scenarios, the low-altitude airspace will be populated with a wide variety of vehicles, including delivery drones, electric vertical takeoff and landing (eVTOL) aircraft, and manned and unmanned aerial vehicles (UAVs), all of which require high-speed, reliable connectivity [2,3]. This surge in low-altitude communications exposes the limitations of conventional terrestrial networks, particularly in terms of airspace coverage, highlighting the urgent need for novel mobile networks capable of providing ubiquitous connectivity to aerial platforms. In response, space-air-ground integrated low-altitude aerial vehicular networks (SAG-LAAVN) emerge as a transformative solution to provide ubiquitous intelligent connectivity for modern three-dimensional intelligent transportation systems (ITS) through the complementary fusion of heterogeneous networks [[4], [5], [6]]. SAG-LAAVN leverages novel technologies to orchestrate a synergistic collaboration among heterogeneous network layers, elements, and functionalities [7,8]. Consequently, it is capable of delivering robust and intelligent services to support the demanding, high-density, and high-mobility low-altitude traffic scenarios.

As a core of future sixth-generation (6G) mobile networks, SAG-LAAVN is engineered to meet the diverse and stringent requirements of advanced air operations. Specifically, it is expected to facilitate massive communication, efficiently managing the concurrent access demands of high-density, large-scale aerial vehicles in increasingly crowded airspaces [9]. Equally essential is its ability to deliver hyper reliable and low latency communication (HRLLC), which is crucial for the safe and timely operation of aerial vehicles in mission-critical scenarios such as formation flying and emergency response [10]. Ubiquitous connectivity further ensures uninterrupted service across varied geographic and environmental conditions. Moreover, the integration of artificial intelligence (AI) enables the network to evolve toward greater autonomy, dynamically adapting to rapidly changing conditions and complex operational requirements [[11], [12], [13]]. Furthermore, integrated sensing and communication (ISAC) provides high-precision and low-cost sensing capabilities alongside data transmission, which are vital for real-time obstacle avoidance and effective airspace management.

SAG-LAAVN is a large-scale, highly complex, heterogeneous network characterized by widely distributed hardware devices and diverse communication protocols [14]. As such, advanced network management techniques are essential to fully realize its synergistic benefits. One promising approach is the innovative hyper-converged architecture that unifies data, operation, information, and communication technologies (DOICT) [15]. This architecture enables SAG-LAAVN to transcend the limitations of traditional ground-based systems while enhancing network resilience, extending coverage, and delivering superior service quality across a variety of operational scenarios. Moreover, it leverages data-driven AI methodologies alongside advanced operational strategies based on digital twins, enabling real-time optimization of network configuration and resource allocation while dynamically adapting to evolving network topologies and user demands [16].

The practical impact of the SAG-LAAVN is substantial, promising to empower a new wave of applications, from large-scale autonomous drone logistics and resilient public safety networks in smart cities to providing critical connectivity in post-disaster emergency scenarios. Nevertheless, fully realizing the potential of SAG-LAAVN will require addressing complex challenges related to three-dimensional global coverage, intelligent network management, and efficient resource coordination among heterogeneous systems.

In this paper, we introduce the SAG-LAAVN, a network system built on the DOICT hyper-converged architecture that provides ubiquitous intelligent connectivity for three-dimensional ITS. First, we present the SAG-LAAVN, which is organized into three segments: space, aerial, and ground. Next, we provide an in-depth analysis of the DOICT architecture, detailing the division of labor among its three technology modules, namely information and communication technology (ICT), data technology (DT), and operational technology (OT), and demonstrating how their coordinated integration enhances network performance. Additionally, we outline three potential technologies: intelligent networking approach, multi-dimensional resource management, and virtual-physical dynamic collaboration. Finally, we present a case study in which SAG-LAAVN is implemented via a fully decoupled radio access network (FD-RAN) [17], thereby demonstrating its ability to deliver ubiquitous, high-quality services. The main contributions of this paper are as follows:

• We design the SAG-LAAVN architecture with FD-RAN as an innovative model, enhancing the flexibility of space-air-ground convergence, the efficiency of resource management, and the intelligence of network services.
• We propose a novel hyper-converged architecture by deeply integrating ICT, DT, and OT, which provides new methodologies and technical references for building secure and intelligent low-altitude networks.
• We conduct simulations and scenario-based validations to evaluate the proposed technologies, demonstrating clear advantages in communication and sensing while confirming the feasibility and effectiveness of SAG-LAAVN for practical deployment.

The remainder of this paper is organized as follows. Section 2 presents an overview of the SAG-LAAVN. Its key challenges are explored in Section 3, and three potential technologies are discussed in Section 4. Section 5 presents a case study, followed by the conclusion in Section 6.

## 2 DOICT hyper-converged SAG-LAAVN

**Fig. 1:**
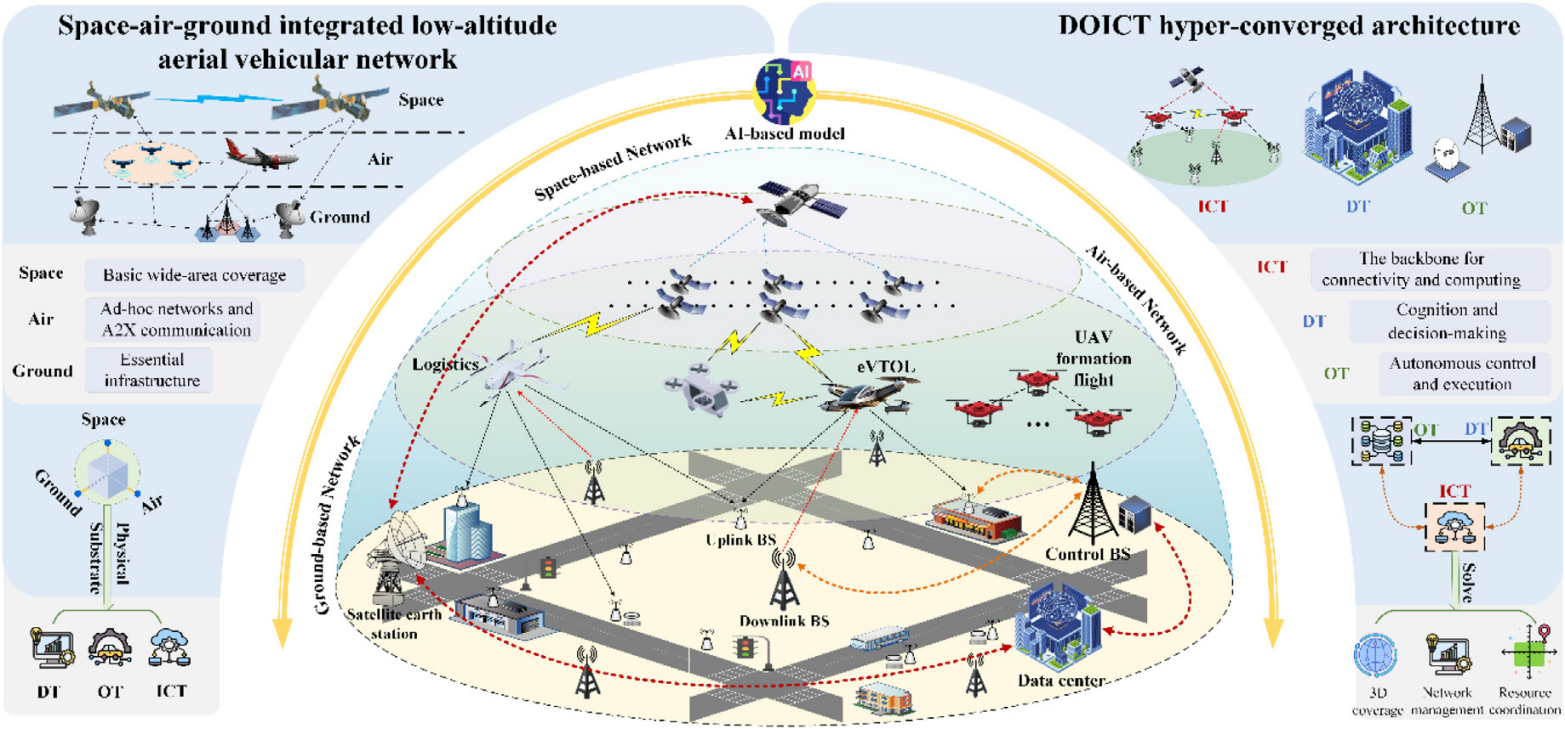
**Overview of space-air-ground integrated low-altitude aerial vehicular networks (SAG-LAAVN), illustrating the integrated physical architecture and the logical DOICT framework**.

As shown in Fig. 1, the SAG-LAAVN leverages the DOICT hyper-converged architecture to provide ubiquitous intelligent connectivity. In this converged network, ICT forms the essential connectivity backbone, DT endows the network with data-driven intelligence, and OT ensures real-time, autonomous operation. Together, the convergence of ICT, DT, and OT under the DOICT architecture creates a synergistic environment that enhances network performance, resilience, and adaptability, attributes that are critical for supporting complex mission-critical applications in three-dimensional ITS.

### 2.1 ICT in SAG-LAAVN

At the core of SAG-LAAVN lies the ICT module, which encompasses both communication technology (CT) and information technology (IT), forming the essential backbone for global connectivity. IT provides comprehensive computing and software infrastructure that orchestrates network functions, such as network virtualization, cloud-native service, and advanced cybersecurity [18]. Meanwhile, CT is tasked with establishing high-speed, reliable communication links via advanced wired and wireless networks, ensuring seamless data transmission across the space, air, and ground segments. Together, IT and CT create a stable and flexible infrastructure where information flows seamlessly and reliably between disparate network nodes and real-time network control and optimization are performed.

In the space segment, the satellite networks consisting of a large number of low earth orbit (LEO) satellites provide global wide-area coverage, extending connectivity to remote areas with insufficient ground infrastructure or to underserved airspace [19]. This ensures continuous high-speed communications even as aerial vehicles operate at varying altitudes or traverse extensive spatial regions.

The aerial segment consists of aerial Ad-hoc networks and aircraft-to-everything (A2X) communications, which are engineered to establish flexible connectivity among neighboring nodes in complex and highly dynamic low-altitude environments. It facilitates real-time data exchange, enhances situational awareness, and supports formation coordination among densely populated aerial vehicles, thereby ensuring safe and reliable operations.

The ground segment integrates the existing terrestrial cellular network with numerous data and computing centers, which together provide foundational support for SAG-LAAVN. It delivers basic coverage for most low-altitude airspace while its core network ensures comprehensive connectivity across all segments. Additionally, the ground segment enables intelligent network management through advanced DT and OT, optimizing overall system performance.

### 2.2 DT in SAG-LAAVN

DT is SAG-LAAVN’s cognitive engine that empowers the network with data-driven intelligence to improve network performance and user experience. DT is tasked with collecting, processing, storing, and analyzing large amounts of data generated by network operations, user interactions, and environmental sensors. By leveraging big data analytics and machine learning algorithms, DT is able to extract actionable insights, predict network behavior, and optimize resource allocation in real-time. In addition, DT facilitates the development of digital twin models that replicate network operations in virtual environments, enabling continuous monitoring, simulation, and proactive management of network performance. This cognitive module not only supports predictive maintenance and dynamic service adaptation, but also lays the foundation for the continuous evolution of the network, enabling it to scale in three dimensions to meet future demands.

### 2.3 OT in SAG-LAAVN

OT serves as the autonomous control center within SAG-LAAVN, ensuring real-time responsiveness and high reliability in managing the network infrastructure. It monitors operational activities across the network and provides rapid feedback to facilitate adaptive decision-making. OT implements advanced automation protocols and leverages real-time analytics to support dynamic reconfiguration of network resources, which is critical to maintaining service continuity under dynamic conditions. In addition, OT incorporates robust safety measures and fault-tolerant mechanisms to identify and resolve potential problems in a timely manner. Through continuous monitoring, proactive management, and adaptive control, OT plays a crucial role in sustaining the overall efficiency, robustness, and resilience of SAG-LAAVN, enabling it to meet the demanding requirements of three-dimensional ITS.

## 3 Key scientific challenges

### 3.1 Seamless three-dimensional coverage

Ensuring seamless three-dimensional coverage of low-altitude aerial vehicles is a core scientific challenge in SAG-LAAVN [20,21]. Traditional terrestrial networks are often unable to provide continuous service in remote areas and are generally unable to provide complete coverage in low-altitude airspace. This requires the fusion of space, air, and ground segments and the development of an efficient collaboration mechanism between heterogeneous networks to achieve complete, multi-layered coverage. In addition, the inherent high mobility of these aerial vehicles, such as long-distance delivery drones, induces large-scale and rapid topology changes, requiring dynamic adjustment of network parameters based on real-time user locations. Another critical issue lies in maintaining reliable and stable connectivity in complex electromagnetic environments, as high mobility introduces significant Doppler shifts. Addressing these issues is essential for delivering uninterrupted, high-quality communication that supports the emerging low-altitude transportation ecosystem.

### 3.2 Efficient resource coordination

Efficient network-wide resource collaboration is the third scientific challenge in SAG-LAAVN, and is critical for optimizing resource utilization and enhancing user experience in large-scale heterogeneous networks [22,23]. Achieving this goal requires coordinated resource allocation capable of efficiently distributing computing and communication resources across space, air, and ground segments to fully utilize resources. Cross-domain scheduling mechanisms also need to be developed to provide on-demand service capabilities that can adapt to diverse real-time user demands, such as simultaneously supporting an uplink-heavy remote sensing task and a downlink-heavy video stream. In addition, a high-fidelity simulation platform is critical to SAG-LAAVN’s system performance assessment to provide accurate insights into network collaboration and guide its continuous improvement. Collectively, these strategies establish a comprehensive coordination mechanism that addresses the scientific challenge of ensuring efficient network-wide collaboration in SAG-LAAVN.

### 3.3 Intelligent network management

Intelligent network management represents another scientific challenge critical to the operation of SAG-LAAVN, particularly given the complexity of operating its space, air, and ground segments. Addressing this challenge requires the design of an advanced network architecture that integrates heterogeneous networks and ensures seamless integration between different communication protocols. Equally important is the efficient scheduling of resources through software-defined networking (SDN) and AI algorithms, which enables the adaptive allocation of multi-dimensional resources based on the dynamic demands, such as clearing an air corridor for an emergency medical eVTOL [24]. In addition, such large-scale complex networks require continuous monitoring mechanisms and fault analysis algorithms for robust fault management and overall network stability to ensure stable data transmission and quick self-healing capabilities. Together, these measures form an intelligent management approach to overcome the operation and maintenance challenges of large-scale complex networks.

## 4 Potential technologies

This section investigates three foundational pillars of potential technologies for realizing SAG-LAAVN. The first pillar, intelligent three-dimensional networking, leverages architectural innovations such as FD-RAN to achieve flexible and adaptive control. The second, multi-dimensional resource management, employs AI-driven approaches to efficiently orchestrate heterogeneous resources. The third, virtual–physical dynamic collaboration, integrates digital twins with advanced sensing technologies to enable proactive and adaptive network evolution. Collectively, these pillars constitute a cohesive framework for building a robust, scalable, and intelligent network.

### 4.1 Intelligent three-dimensional networking

**Fig. 2:**
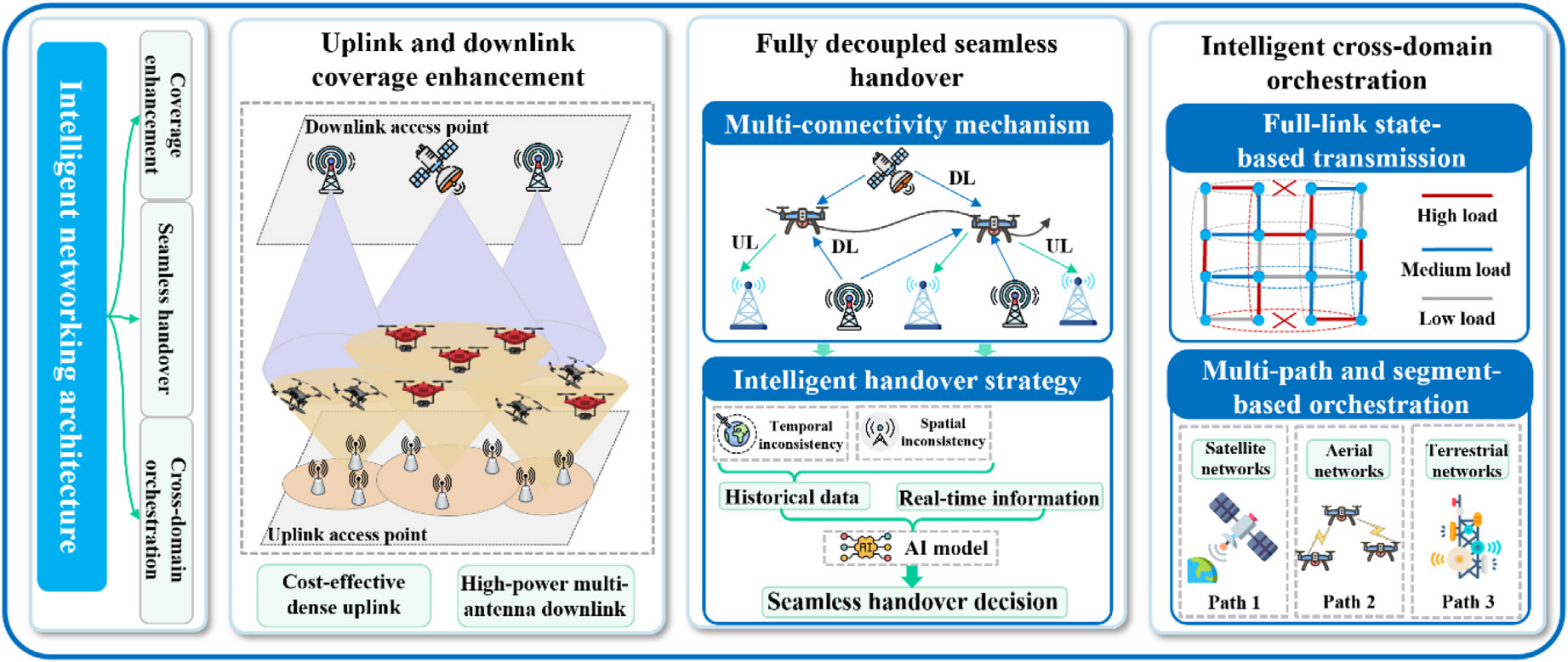
**Intelligent networking approach for space-air-ground integrated low-altitude vehicular networks**.

The intelligent networking approach is shown in Fig. 2, which encompasses network coverage enhancement, seamless handover mechanism, and intelligent cross-domain orchestration, enabling efficient and reliable connectivity in dynamic SAG-LAAVN.

1) *Uplink and downlink coverage enhancement*: In the FD-RAN-enabled SAG-LAAVN, enhancing uplink and downlink coverage is essential for ensuring reliable connectivity and efficient service delivery. FD-RAN decouples a traditional base station into three types of base stations: uplink base station, downlink base station, and control base station. For the uplink, dense deployment of low-cost access points can be utilized to support massive concurrent transmissions from low-altitude aerial vehicles, thereby improving network accessibility and reducing transmission delay. For the downlink, high-power access points equipped with multi-antenna techniques can be employed to provide wide-area coverage and stable high-rate transmission, meeting the stringent quality-of-service requirements of aerial applications.
2) *Fully decoupled seamless handover*: In the SAG-LAAVN, seamless handover is essential to ensure service continuity in highly dynamic aerial environments. A fully decoupled multi-connectivity mechanism allows low-altitude aerial vehicles to maintain simultaneous uplink and downlink associations with multiple heterogeneous access points, such as terrestrial base stations and satellites, thereby reducing the risk of link interruptions [25]. To further enhance robustness, an intelligent handover strategy leverages historical data, such as satellite orbits and terrestrial base station distributions, along with real-time information to mitigate temporal and spatial inconsistencies. By employing AI-driven models to fuse multi-source information, the system can make adaptive and seamless handover decisions, ensuring uninterrupted connectivity and consistent service quality.
3) *Intelligent cross-domain orchestration*: In the SAG-LAAVN, intelligent cross-domain orchestration is crucial for optimizing the management of heterogeneous network infrastructures. Full-link state awareness enables dynamic assessment of network load and vulnerability points, allowing proactive control of data for efficient cross-domain transmission. Moreover, orchestration strategies based on multi-path and space-air-ground segmentation leverage the unique capabilities of different network domains, including satellite networks for wide-area coverage, aerial networks for flexible deployment, and terrestrial networks for high-capacity access. By intelligently integrating these diverse communication paths, the SAG-LAAVN can achieve robust and resilient connectivity that adapts to dynamic operational requirements and environmental variations.

### 4.2 Multi-dimensional resource management

**Fig. 3:**
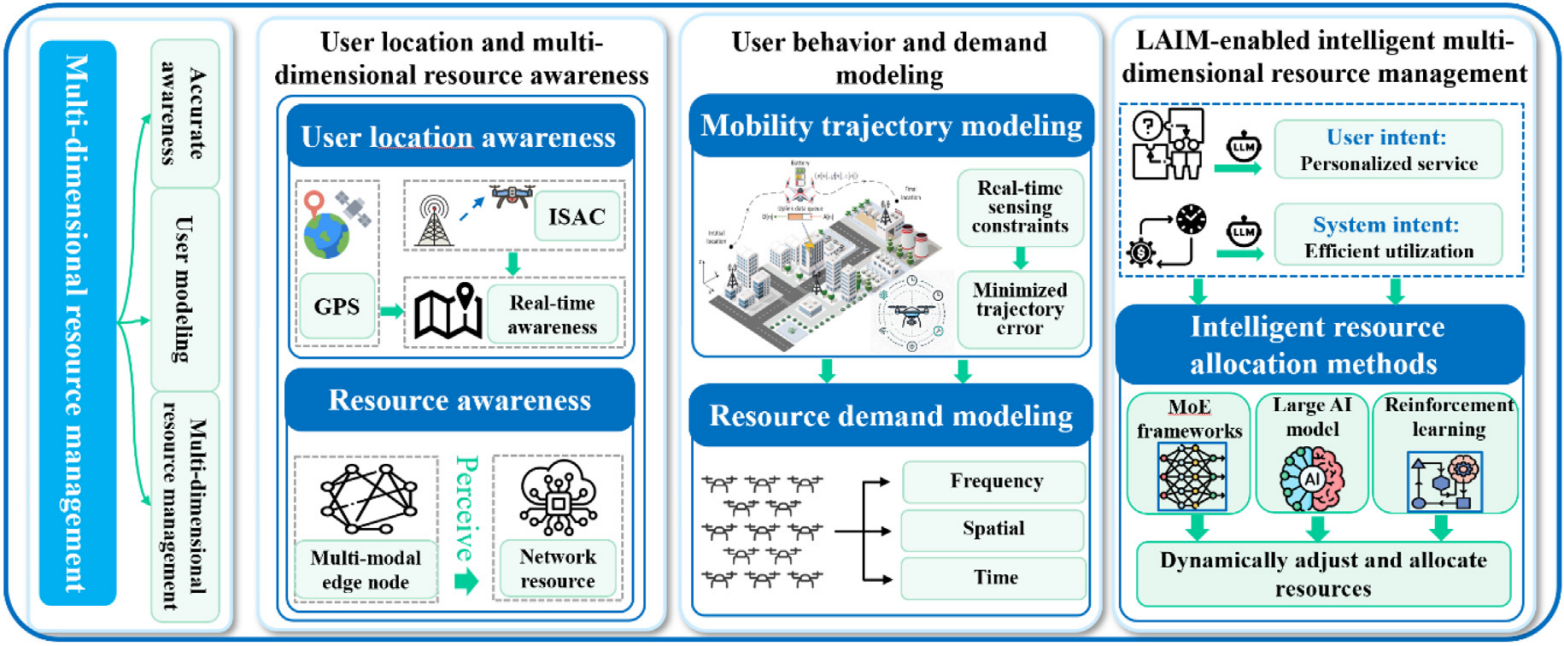
**Multi-dimensional resource management for space-air-ground integrated low-altitude vehicular networks**.

The multi-dimensional resource management is shown in Fig. 3, which encompasses user and resource awareness, user modeling, and intelligent multi-dimensional resource management, improving the operational efficiency of complex SAG-LAAVN.

1) *User location and multi-dimensional resource awareness*: Precise location awareness of aerial vehicles and comprehensive awareness of network resources are essential for efficient network operation [[26], [27], [28]]. The advent of ISAC technologies enables simultaneous data transmission and user perception [29]. Furthermore, deploying multi-modal sensors and data analytics algorithms on edge servers facilitates effective cross-node aggregation and monitoring of network resource states. This integrated approach establishes real-time situational awareness of multi-dimensional network resources, including spectrum, sensing, computation, and storage.
2) *User behavior and demand modeling*: In SAG-LAAVN, accurate modeling of user behavior and service demands is critical for efficient multi-dimensional resource management. On one hand, mobility trajectory modeling accounts for real-time sensing constraints and trajectory planning to minimize path deviations, thereby ensuring precise behavioral modeling of low-altitude aerial vehicles. On the other hand, resource demand modeling captures the multi-dimensional characteristics of service requests by constructing a demand matrix across frequency, spatial, and temporal dimensions [30]. Such modeling provides a fine-grained description of heterogeneous service requirements, enabling the network to allocate resources more efficiently and adaptively.
3) *LAIM-enabled intelligent multi-dimensional resource management*: In SAG-LAAVN, an intelligent resource management approach based on a large AI model (LAIM) is essential for achieving dynamic and precise matching between the complex demands of large-scale users and the network’s multi-dimensional resources [[31], [32], [33]]. Initially, LAIM has to comprehend user intentions, such as flight trajectories and communication requirements, to enable personalized service delivery. Simultaneously, it has to understand the system’s objectives, such as the efficient utilization of overall network resources. By leveraging advanced multi-modal large language model (LLM) together with mixture-of-experts frameworks and reinforcement learning techniques, LAIM can intelligently optimize the orchestration of heterogeneous resources, including communication, sensing, computation, and storage [34].

### 4.3 Virtual-physical dynamic collaboration

**Fig. 4:**
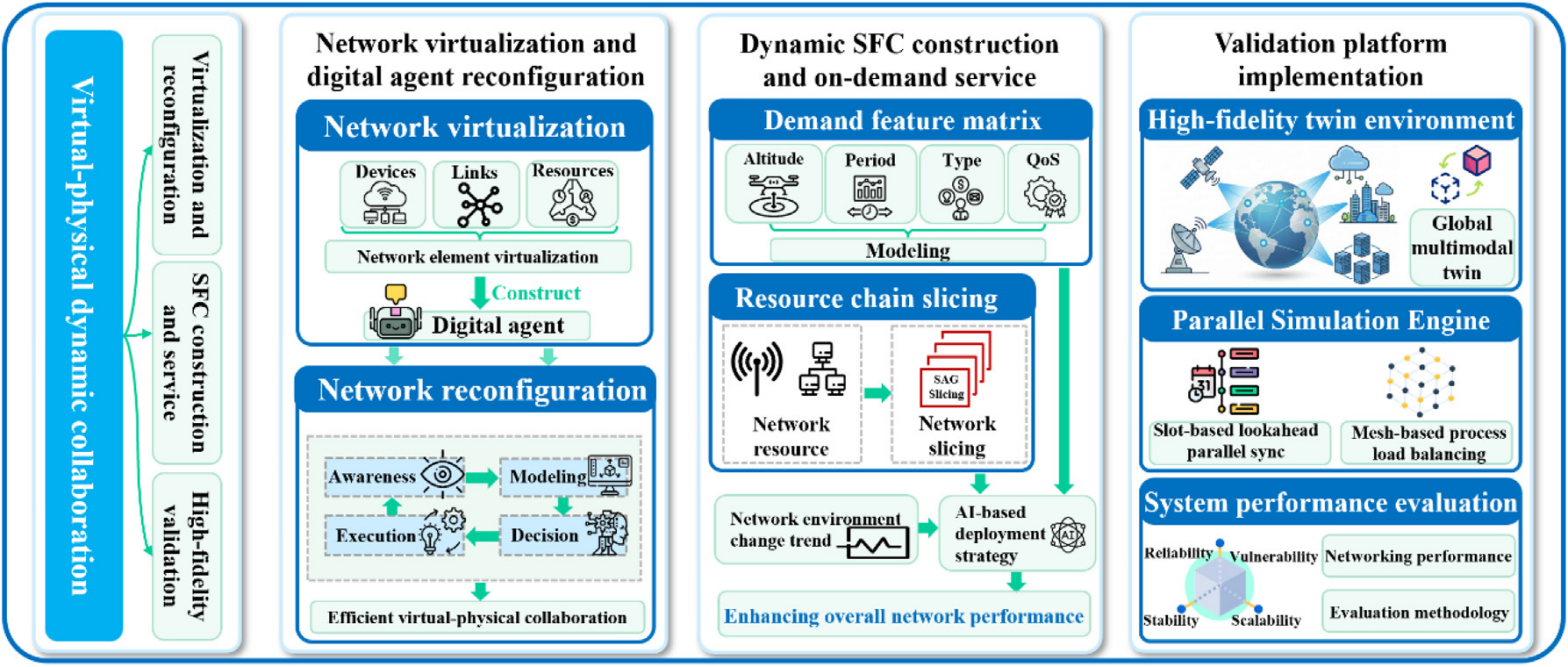
**Virtual-physical dynamic collaboration for space-air-ground integrated low-altitude aerial vehicular networks**.

The virtual-physical collaboration is shown in Fig. 4, which encompasses network virtualization and reconfiguration, service function chain (SFC) construction and on-demand service, as well as validation platform implementation, enhancing the collaboration performance.

1) *Network virtualization and digital agent reconfiguration*: The combination of network virtualization and digital twin technology enables the construction of digital agents, which in turn enables dynamic reconfiguration of the network based on digital agents [35,36]. This process begins by virtualizing various network elements to construct their corresponding digital twins [37]. These twins are then augmented with advanced intelligence, increasingly driven by recent breakthroughs like generative AI, to evolve them into proactive digital agents. These digital agents operate within a closed-loop evolutionary system that involves extracting information from network elements, updating the state of the digital agents, making reconfiguration decisions based on current network conditions, and managing the network through the SDN controller.
2) *Dynamic SFC construction and on-demand service*: The construction of SFC in SAG-LAAVN faces dual challenges of resource fragmentation and highly diverse user demands. By leveraging digital agents, virtual network resources can be integrated and sliced according to their characteristics. A demand feature matrix for low-altitude aerial services can be modeled across multiple dimensions, including spatial altitude, temporal cycles, functional types, and quality of service. This enables efficient fulfillment of diverse requirements after SFC deployment. Furthermore, the virtualized digital agents can be used to predict network dynamics, while AI-based strategies dynamically adjust SFC placement based on the demand feature matrix, thereby achieving personalized and on-demand services [38].
3) *Validation platform implementation*: To rigorously validate the complex SAG-LAAVN, it is necessary to design and implement a comprehensive validation platform [39,40]. Built upon a high-fidelity twin environment, the platform constructs a global multimodal twin that accurately mirrors the complexity of the physical world. A parallel simulation engine powers the platform, employing slot-based lookahead synchronization and mesh-based process load balancing to enable efficient, large-scale simulations. Furthermore, the platform facilitates holistic system-level performance evaluation by assessing key metrics, including reliability, stability, scalability, and vulnerability, through a structured evaluation methodology.

## 5 Case study

This section presents the system model and simulation parameters of the SAG-LAAVN implemented via the FD-RAN. The simulation results are then presented with a detailed analysis.

### 5.1 System model and simulation setup

The proposed SAG-LAAVN is implemented via the FD-RAN [41,42]. In FD-RAN, uplink and downlink transmissions are processed by separate base stations connected to the edge cloud. This uplink-downlink decoupled design enables flexible, seamless coordination under heterogeneous SAG-LAAVN conditions, providing tailored services to aerial vehicles. Specifically, aerial vehicles can freely choose between ground base stations (GBSs) and LEO satellites for uplink and downlink transmissions without the constraint of using the same node for both directions. Crucially, the adaptability of FD-RAN demonstrated in our simulations is unlocked and driven by the DOICT architecture. It incorporates innovative ICT, such as multi-GBS joint transmission, while leveraging DT by using historical data to model the network and optimize transmission parameters. Moreover, by facilitating control-data plane separation, FD-RAN provides the perfect infrastructure for OT to execute dynamic, real-time control, thereby efficiently harnessing the full intelligence of the DOICT framework within SAG-LAAVN.

**Fig. 5:**
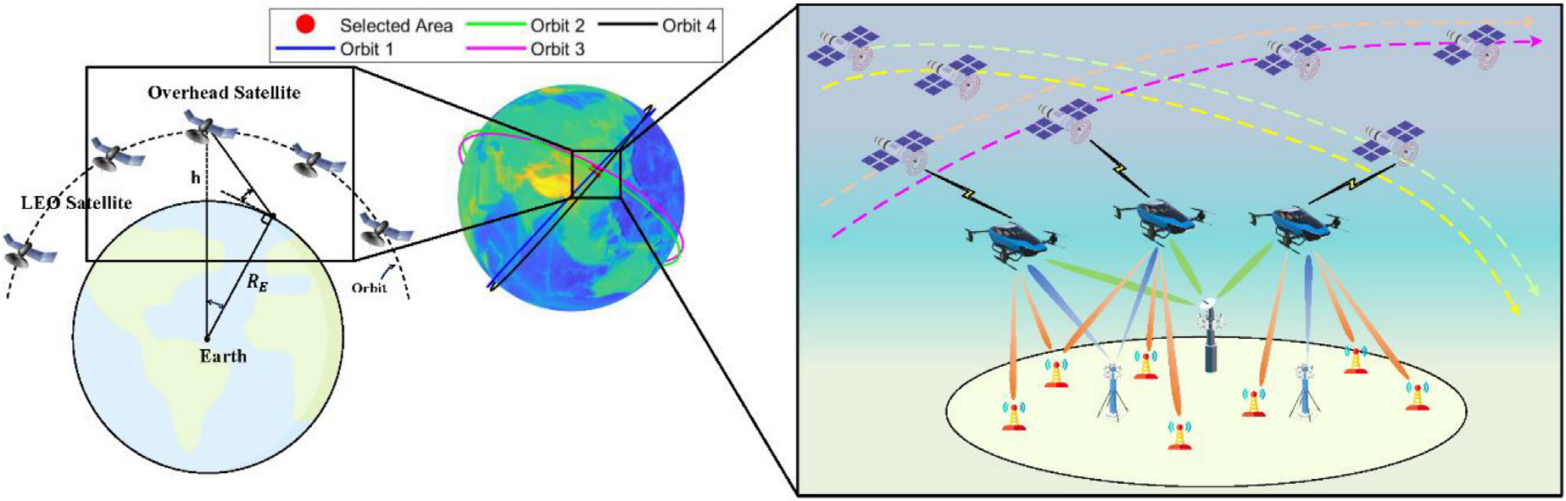
**The system model illustration of space-air-ground integrated low-altitude aerial vehicular networks**.

As shown in Fig. 5, we consider a SAG-LAAVN scenario with K GBSs and N satellites to provide service to aerial vehicles. All GBSs, satellites, and aerial vehicles are equipped with a single antenna. For the terrestrial link, the channel gain between the aerial vehicle and each GBS is governed primarily by carrier frequency and link distance, and the per-link SNR follows from the transmit power and noise; signals from the serving GBS set are then combined using maximal ratio combining (MRC) to yield the aggregate SNR. For the satellite link, large-scale fading is modeled as the sum of free-space path loss, ground-clutter loss, atmospheric/gaseous absorption, rain attenuation and cloud scattering/absorption; each term is parameterized according to ITU recommendations and depends on frequency, link geometry (elevation and distance) and meteorological variables (pressure, temperature, rain rate and liquid-water density) [43].

The simulation setup for SAG-LAAVN is as follows. Nineteen GBSs are deployed with a spacing of 650 m, and 4 satellites traverse orbits at an altitude of 500 km. The aerial vehicle transmit power is set as $P_{u}=20$ dBm, the GBS transmit power is set as $P_{b}=35$ dBm, the satellite transmit power is $P_{d}=98$ dBm [44]. The noise power is fixed as $n_{0}=-105$ dBm, and all other coefficients are specified according to ITU-R recommendations.

### 5.2 Communication performance analysis

**Fig. 6:**
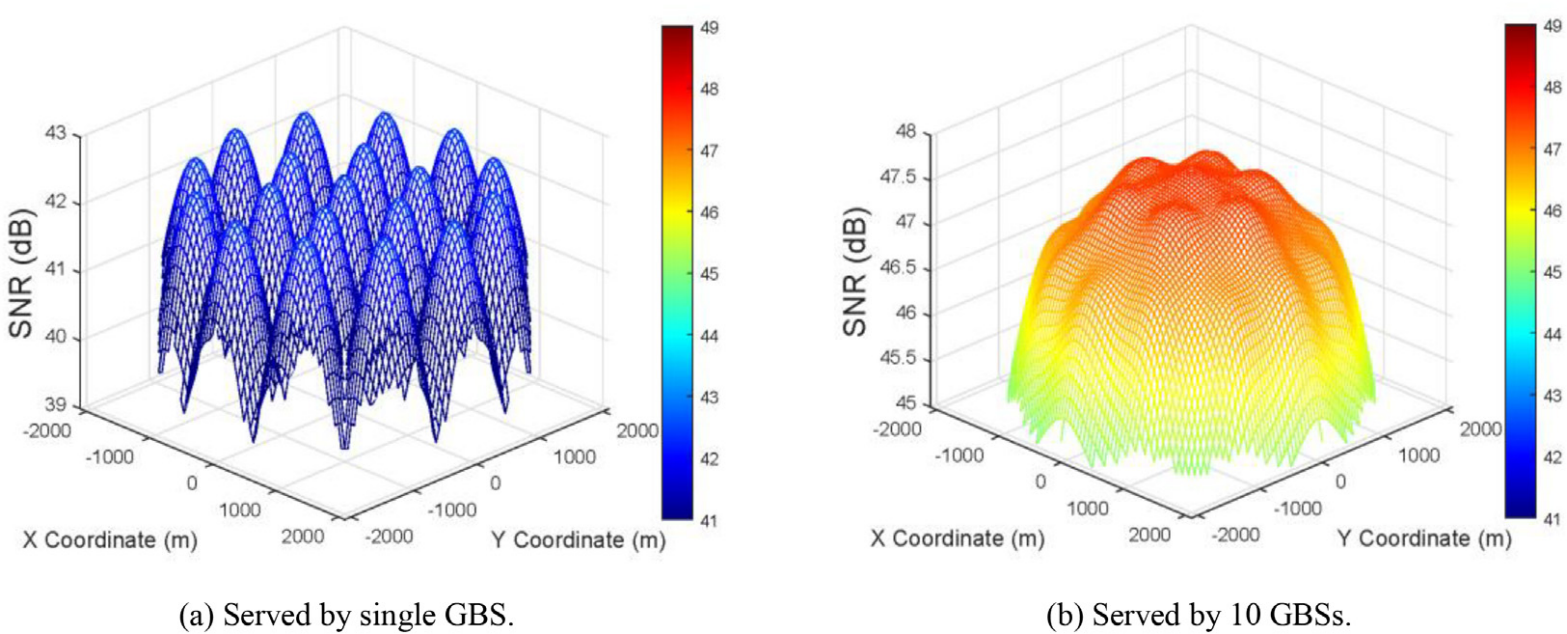
**SNR map at altitudes of 600 m with different receiving GBSs**.

Due to the fixed location of the GBS, the terrestrial network’s coverage of low-altitude airspace exhibits significant spatial inconsistency. Fig. 6 shows the coverage performance at the altitude of 600 m, where the aerial vehicle selects the nearest single GBS or the nearest 10 GBSs, respectively. As depicted by sharp peaks and valleys in Fig. 6a, using a single GBS yields a high SNR directly above the GBS but causes a rapid drop at the cell edge. The comparison between Fig. 6a,b shows that employing multiple GBSs with MRC creates a much smoother and wider coverage area, which substantially improves overall performance. Notably, multi-GBS collaboration can significantly enhance the SNR at the airspace above the cell edge and maintain stable connectivity for aerial vehicles. In summary, joint service from multiple GBSs is an effective strategy for improving terrestrial network coverage in low-altitude airspace.

**Fig. 7:**
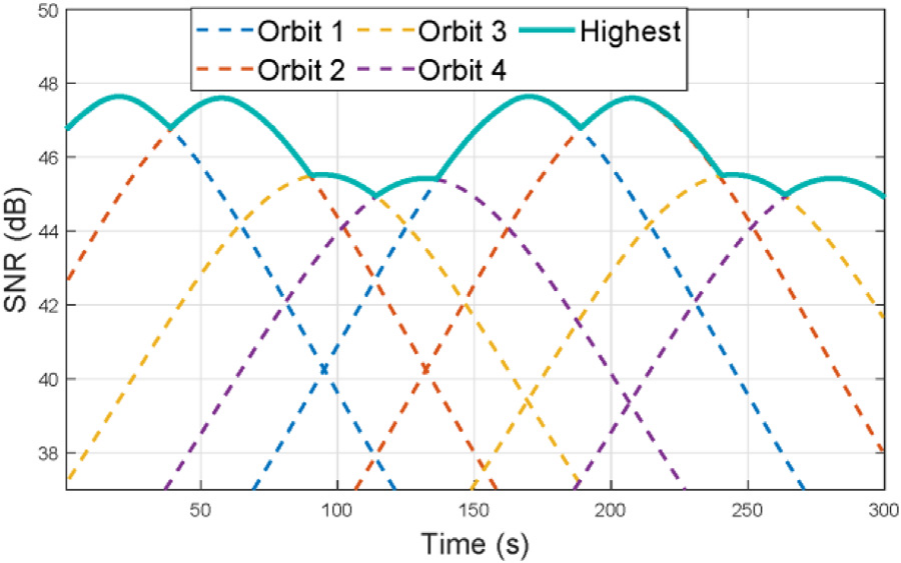
**SNR for satellite networks over time**.

Regarding satellite coverage, the swift movement of satellites results in rapid variations in the distance between the aerial vehicle and each satellite, leading to pronounced time-dependent fluctuations in SNR. The movement of aerial vehicles is relatively negligible compared to the movement of satellites, so satellite coverage primarily exhibits temporal inconsistency. Fig. 7 shows the SNR variations over time: the dashed lines of the same color represent the SNR provided by different satellites on the same orbit, while the solid line shows the maximum SNR that could be achieved if the vehicle is continuously connected to the nearest satellite. The results show that the SNR of the satellite link follows a periodic pattern that reflects the orbital trajectory of satellites. When multiple satellites are simultaneously available, a flexible handover mechanism allows the aerial vehicle to consistently select the satellite with the strongest signal at any given moment.

**Fig. 8:**
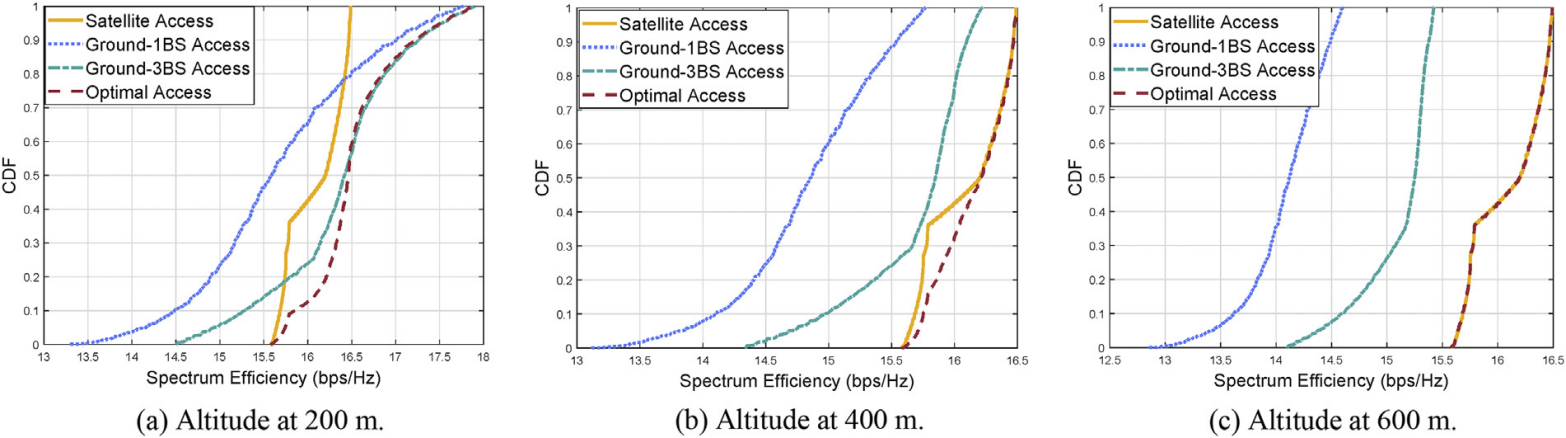
**Comparison of downlink spectral efficiency for different transmission schemes at various altitudes**.

Spectral efficiency is a critical performance metric for wireless networks and is employed here to further evaluate SAG-LAAVN’s performance. Fig. 8 shows the cumulative distribution function (CDF) of spectral efficiency for different access approaches across various altitudes during downlink transmission. Specifically, compared with uplink transmission, due to the higher deployment costs associated with downlink GBSs and the increased complexity of joint downlink transmission, we consider a configuration where downlink services are provided by at most 3 GBSs. Optimal access is achieved by dynamic selection of the node with the highest spectral efficiency. The results in Fig. 8 reveal that the multi-GBS with MRC achieves substantially higher spectral efficiency than single-GBS, with this advantage increasing at higher altitudes. In contrast, the range of spectral efficiency values provided by satellite network remains relatively narrow and largely independent of altitude. In the lower-altitude case shown in Fig. 8a, satellite network only surpasses ground network at the cell edge, whereas at 400 m altitude shown in Fig. 8b, satellite network delivers higher spectral efficiency over a wider coverage area, with ground network serving as a supplement where satellite signals weaken. At 600 m altitude shown in Fig. 8c, satellite network outperforms ground network in all regions and the optimal access provided by satellites only. In summary, optimal spectral efficiency can be achieved by flexibly choosing access nodes based on the height and distance from GBSs.

**Fig. 9:**
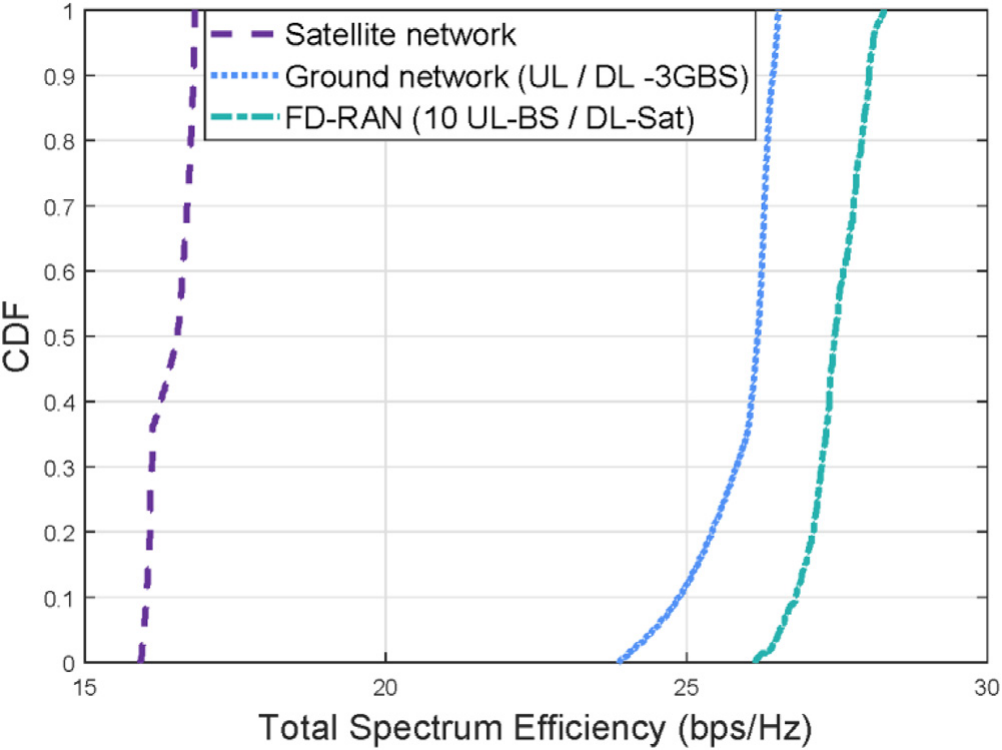
**Spectral efficiency comparison for different networks at 600 m**.

For SAG-LAAVN based on FD-RAN and DOICT architecture, the aerial vehicle has the flexibility to select any nodes, either from the GBSs or satellites, for uplink and downlink transmissions. For instance, given the limited power of aerial vehicles and lower path loss, the uplink data can be transmitted through the ground network, where signals from multiple uplink GBSs with lower deployment costs can be jointly processed to enhance the SNR. The comparison of spectral efficiency for different access schemes is demonstrated in Fig. 9. The three curves correspond to different transmission schemes and access networks: (1) coupled satellite network with both uplink and downlink transmissions through satellite network, (2) coupled ground network with both uplink and downlink transmissions through ground network, and (3) FD-RAN with dynamic selection of the node with the highest spectral efficiency for uplink and downlink. As can be seen, the satellite network shows the worst performance due to the considerably lower performance during uplink transmission, although it achieves a higher spectral efficiency than ground network in Fig. 8c. In contrast, FD-RAN provides an optimal spectral efficiency higher than accessing the coupled ground or satellite network alone.

### 5.3 Sensing performance analysis

In this simulation, the aerial vehicle is assumed to follow a circular trajectory with a radius of 1 km. The localization principle is based on the observation that the Doppler frequency shifts, inherently embedded in the uplink OFDM signals, are determined by the relative motion between the aerial vehicle and the GBSs. During the carrier frequency offset (CFO) compensation at each BS, these Doppler components are implicitly extracted and can thus be exploited for localization. By utilizing the estimated Doppler values, a set of equations is constructed, with the vehicle’s position and velocity treated as unknown parameters to be jointly inferred. For each localization instance, the Doppler measurements from the 10 GBSs with the highest received SNRs are selected for aerial vehicle localization.

**Fig. 10:**
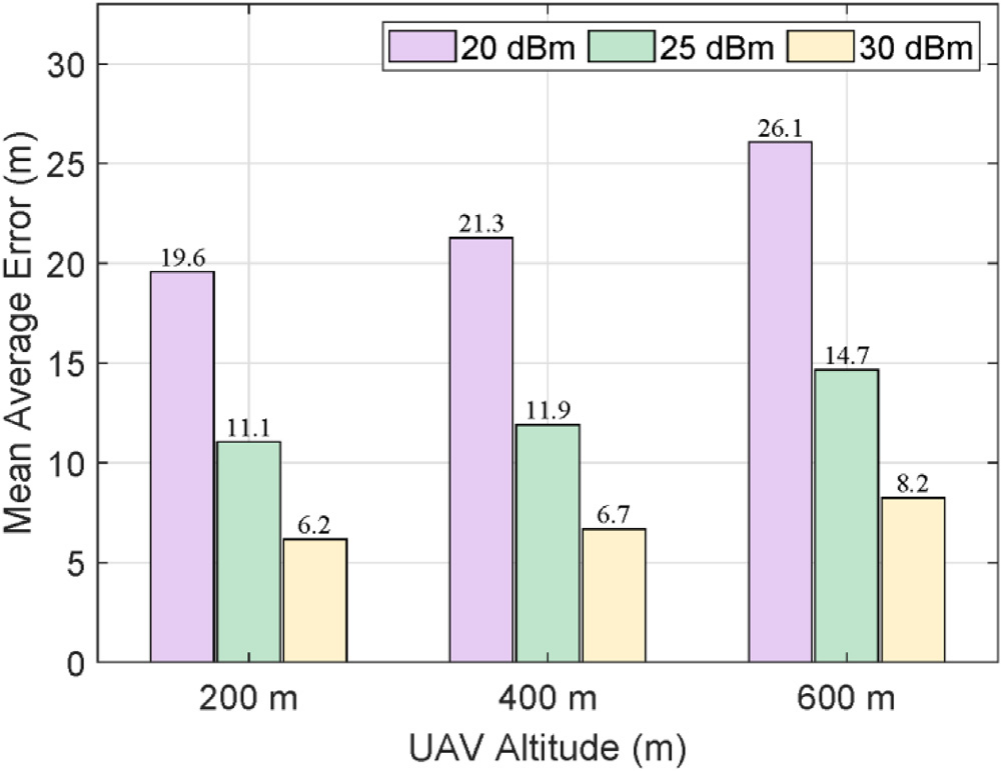
**Localization error comparison for different altitudes and transmission power**.

Fig. 10 presents the mean localization error for an aerial vehicle transmitting at different power levels while circling at varying altitudes. As observed, increasing the transmit power improves the received SNR at the GBSs, thereby enabling more accurate Doppler estimation and reducing localization error. Conversely, at higher altitudes, the increased distance to the GBSs leads to lower SNRs, which degrade Doppler estimation accuracy and result in larger localization errors. These findings demonstrate that cooperative sensing across multiple GBSs can effectively harness Doppler information to realize reliable aerial vehicle sensing, highlighting the feasibility of multi-BS collaboration for UAV detection, localization, and tracking.

## 6 Conclusion

In this paper, we have presented the SAG-LAAVN as a new paradigm to provide ubiquitous intelligent connectivity. By harnessing the innovative DOICT hyper-converged architecture, SAG-LAAVN should meet the critical performance requirements of three-dimensional ITS. The three potential technologies of SAG-LAAVN have been fully discussed. The case study based on FD-RAN has demonstrated the effectiveness of SAG-LAAVN. For future work, we will explore advanced AI-driven mechanisms for SAG-LAAVN, such as multi-agent reinforcement learning for resource orchestration, generative predictive digital twins for proactive management, and security frameworks for robust threat detection and mitigation.

## CRediT authorship contribution statement

**Haibo Zhou:** Conceptualization, Methodology, Data curation, Project administration, Writing – original draft, Writing – review & editing, Supervision. **Jianzhe Xue:** Methodology, Data curation, Writing – original draft. **Yi Yuan:** Methodology, Data curation, Writing – original draft. **Zeyu Sun:** Methodology, Data curation, Writing – original draft. **Yunting Xu:** Conceptualization. **Jiacheng Chen:** Supervision, Writing – review & editing. **Xuemin Shen:** Supervision.

## Declaration of competing interest

The authors declare that they have no conflicts of interest in this work.
